# Immune network dysregulation precedes clinical diagnosis of asthma

**DOI:** 10.1038/s41598-020-69494-x

**Published:** 2020-07-30

**Authors:** Yi-Shin Chang, Benjamin Turturice, Cody Schott, Patricia Finn, David Perkins

**Affiliations:** 1 Division of Pulmonary, Critical Care, Sleep, and Allergy, Department of Medicine, University of Illinois at Chicago College of Medicine, Chicago, IL USA; 2 Department of Bioengineering, University of Illinois at Chicago College of Medicine, Chicago, IL USA; 3 Department of Microbiology and Immunology, University of Illinois at Chicago College of Medicine, Chicago, IL USA; 4 Division of Nephrology, Department of Medicine, University of Illinois at Chicago College of Medicine, Chicago, IL USA; 5 Department of Surgery, University of Illinois at Chicago College of Medicine, Chicago, IL USA

**Keywords:** Transcription, Transcriptomics

## Abstract

Allergic asthma is a chronic disease beginning in childhood that is characterized by dominant T-helper 2 cell activation without adequate counter-regulation by T-helper 1 cell and regulatory T cell activity. Prior transcriptomic studies of childhood asthma have primarily investigated subjects who already have a disease diagnosis, and have generally taken an approach of differential gene expression as opposed to differential gene interactions. The immune states that predispose towards allergic sensitization and disease development remain ill defined. We thus characterize immune networks of asthmatic predisposition in children at the age of 2, prior to the diagnosis of allergic asthma, who are subsequently diagnosed with asthma at the age of 7. We show extensive differences of gene expression networks and gene regulatory networks in children who develop asthma versus those who do not using transcriptomic data from stimulated peripheral blood mononuclear cells. Moreover, transcription factors that bind proximally to one another share patterns of dysregulation, suggesting that network differences prior to asthma diagnosis result from altered accessibility of gene targets. In summary, we demonstrate non-allergen-specific immune network dysregulation in individuals long before clinical asthma diagnosis.

## Introduction

Childhood asthma is a disease of high prevalence^1,2^ that eludes early diagnosis^3^. Diagnosis in young children is challenging due to the lack of specificity in the early clinical presentation of atopy and wheeze. While nearly half of the population will experience at least one wheezing episode in early childhood, most individuals will not go on to develop asthma^3,4^. Spirometry, which is the gold standard for asthma diagnosis, is typically not utilized in children under the age of 6. It is thus important to characterize early immune states that predispose toward clinical diagnosis of asthma in order to facilitate identification of individuals that are poised to develop disease.

The prototypic immune alteration in allergic asthma is that of dominant T-helper 2 cell (Th2) activation without adequate counter-regulation by T-helper 1 (Th1) and regulatory (Treg) cells^5^. The process of allergic sensitization involves differentiation of these effector T-cell populations and re-shaping of their cytokine profiles^6^. Less is known regarding the differential basal immune states that predispose towards allergic sensitization and disease development, “asthmatic poise.” Our laboratory has previously reported altered Th2 cytokine elaboration in response to common aeroallergens in the cord blood mononuclear cells of neonates with differing in utero microbial exposures^7^. These differential immune signatures can be probed as early as birth.

Genome-wide association studies (GWAS) have identified genetic polymorphisms associated with asthma and other atopic phenotypes, but, to date, explain only a small percentage of disease heritability^3^. Explanations for the “missing heritability” include risk from copy number variation, gene–gene interactions, and gene-environment interactions which are not generally assessed in GWAS^5^. Findings of epigenetic alterations in asthma suggest a strong mediatory effect of epigenetic modifications to disease susceptibility^3^. For example, the ratio of histone deacetylase (HDAC) to histone acetylase (HAT) is known to be perturbed by environmental agents such as tobacco smoke^6^, and to correlate with asthmatic status and disease severity^8–14^. The HDAC/HAT ratio has further been shown to influence the Th1/Th2 balance^10–14^. Genome-wide methylation changes have been demonstrated in response to environmental agents^15^, and were identified in a meta-analysis of children who develop asthma^16^.

Investigation of genome-wide expression networks and gene regulatory networks prior to the development of asthma may provide clues of altered gene–gene interactions and epigenetic effects that underlie allergic predisposition. In a prior study, German cockroach extract (CR) stimulation of peripheral blood mononuclear cells (PBMCs) increased natural killer cell-type gene expression in 2-year-olds who developed aeroallergen sensitization by age 3 and clinical asthma by the age of 7^18^. These differentially expressed genes were only found in children with both CR sensitization and asthma by the age of 7. Similar to our findings in a prior study, immune signatures correlated with home allergen levels (CR)^7^. We posit that these differential transcriptomic responses represent early pathways of sensitization to CR. In order to identify non-allergen-specific biomarkers of asthma and to characterize underlying immune states of asthmatic predisposition, in this study we analyze the tetanus toxoid (TT)-stimulated PBMCs from the same study cohort using a network-centric approach. TT stimulation elicits an unbiased and broad immune recall response, since all the children received tetanus vaccination in infancy. It therefore provides a useful immune perturbation, allowing for characterization of more subtly altered immune networks in asthmatic poise prior to clinically diagnosable disease. We elucidate differences of gene expression networks and gene regulatory networks, and infer epigenetic changes, in children at the age of 2 who develop asthma by age 7 compared to those who do not.

## Methods

### Data

Gene expression data was downloaded from the Gene Expression Omnibus database (GSE96783), and consisted of RNAseq data from children enrolled in the Urban Environment and Childhood Asthma (URECA) study, in which subjects have parental history of allergic disease and live in low-income urban areas^17,18^. In this prior study, RNA sequencing was performed on peripheral blood mononuclear cells (PBMCs) from the children at the age of 2, incubated with either German cockroach extract (CR) or dust mite extracts, tetanus toxoid (TT), or media alone (no stimulation). For this study, we utilize RNAseq data from the CR-stimulated, TT-stimulated, and un-stimulated PBMCs, with in-depth analysis of gene expression networks and gene regulatory networks from TT-stimulated data. We compare TT-stimulated networks of children at the age of 2 who developed aeroallergen sensitizations (including CR, dust mite, or both) by the age of 3 and clinical asthma by the age of 7 (asthma, n = 19 with TT data) versus matched subjects who did not have any aeroallergen sensitizations or asthma at age 7 (control, n = 30). Asthma at 7 years of age was defined by a pre-specified algorithm including use of asthma medications in the previous year, spirometry with reversibility, and bronchial hyperresponsiveness assessed using a methacholine challenge. The case group demonstrated a higher incidence of wheezing illnesses and symptoms of atopic dermatitis in the first year of life compared to controls. More details about these subjects and samples including demographic data, home allergen exposure, clinical data, IgE levels, case criteria, and PBMC stimulation and processing are available at the URECA study^17^ and Altman et al.^18^.

### Differential gene expression with CR and TT stimulation

*DESeq2* was used to obtain variance stabilized transformations of raw RNAseq count data^19^, and to perform differential expression analysis. Wald’s test was used to identify genes that changed expression after CR stimulation (compared to no stimulation) and after TT stimulation, separately for controls (n = 30) and asthma (n = 19). Wald’s test was also used to assess for interactions between group and stimulation. Significance of differential expression was assessed at *p* < 0.05 with false discovery rate (FDR) correction.

### Gene expression modules

For the 5667 genes that were determined to be perturbed by TT stimulation (q < 0.05) in either asthma or controls, weighted gene correlation network analysis (WGCNA)^20^ was used to identify modules of highly correlated genes. WGCNA was performed with a soft thresholding power of 12 to produce scale-free network topology, a signed network and topology overlap matrix, the default minimum module size of 30 genes, and a cut height of 0.15. Gene ontology enrichment analysis was performed using the PANTHER classification system^21,22^ and MsigDB^23,24^ to characterize the biological processes captured by each gene expression module. Downstream analyses of gene expression modules were performed on the modules which had significant functional enrichments.

Module eigengenes (first principal component) were calculated for each module for the control expression data with no stimulation and with TT stimulation, and for asthma expression data with no stimulation and with TT stimulation. Two-way ANOVAs with repeated measures were used to identify group x TT-stimulation interaction effects of each module eigengene.

To identify high centrality genes in the WGCNA modules, a protein–protein interaction network was constructed for each module based on the STRING database (https://string-db.org). Default parameters were used to construct the gene interaction networks, and the *igraph* package in R was used to calculate centrality for each gene in the network^25^.

### Network connectivity of gene expression modules

Pairwise connectivity of WGCNA modules was calculated using Pearson correlations of module eigengenes, separately for controls and asthma. Statistically significant associations were assessed at *p* < 0.05 separately for each group with FDR correction.

### Regulatory network construction using PANDA

In order to examine regulatory differences that might give rise to altered WGCNA module connectivity, PANDA (Passing Attributes between Networks for Data Assimilation)^26^ was used to construct regulatory networks separately for the control and asthma group, and the no-stimulation and TT stimulation condition (four networks total). For each PANDA model, the transcriptomic matrix included the expression data from genes that were included in the WGCNA analysis, as well as expression of transcription factors with binding profiles in the JASPAR^27^ database (n = 338, listed in Table S1). In order to obtain an initial regulatory network, we used a motif-based transcription factor (TF) mapping to genes included in our transcriptomic matrix. TF position frequency matrices (PFMs) were obtained from JASPAR, and were mapped to the promoter regions of each gene from 1000 base pairs upstream of the transcriptional start site to 200 base pairs downstream. A motif match of 80% maximum accuracy was counted as a TF-target “hit,” and the number of hits of each TF with each target was used an initial input regulatory network to PANDA. The initial protein–protein interaction network was derived from the STRING database interaction scores between all TFs used in our initial regulatory network.

### Patterns of TF regulatory shifts

Regulatory strength (z scores outputted from PANDA in the regulatory network) of each TF was compared between asthma and controls. Due to the observation that most TFs demonstrated a combination of stronger and weaker target regulation depending upon target module membership, regulatory shift was calculated for each TF with each module. For example, the regulatory shift of GATA3 on CREM, one of its targets in the Th2 module, is simply z_asthma,GATA3 -> CREM_ - z_control,GATA3 -> CREM_. The regulatory shift of GATA3 on Th2, then, was calculated as the median shift of GATA3 across all of its gene targets in the Th2 module. TFs with at least one target in each module were then clustered using k-means based upon regulatory shift across WGCNA modules. A Euclidean distance metric was used, with number of clusters ranging from 2 to 10, each time calculating mean Silhouette score of the clustering result in order to assess clustering quality and to obtain the optimal number of TF clusters. The TF clusters obtained from this analysis are subsequently referred to as TF regulatory clusters.

### Binding locations of TFs in differentially methylated regions

The proximity of binding locations between TFs (based on motif data) was assessed in differentially methylated regions (DMRs) identified from a separate meta-analysis. In this prior meta-analysis of cord blood mononuclear cells (CBMCs) from neonates who are eventually diagnosed with asthma, 35 DMRs were identified^16^. Of these 35 DMRs, ten were in genes that changed expression with TT stimulation in our cohort. We assessed the similarity of binding locations in these 10 DMRs of TFs within the same regulatory cluster compared to TFs in different regulatory clusters. The specific methodology is described below.

For each of the 10 DMRs, binding locations of TFs on DMRs were identified in the same manner as described for the PANDA analysis above, with motif-based TF mapping. The similarity of binding location within versus between TF regulatory clusters was then assessed by computing a distance matrix of binding distances between every pair of TFs, and then calculating Silhouette score based upon TF regulatory cluster membership. This procedure was executed as described in the following steps:

1. The binding level of TF_i_ is represented as a vector of length n_DMR_, where n_DMR_ = the # of bp in the DMR, i = 1:m_DMR_, m_DMR_ = # of TFs that bind on the DMR.
2. For each location along the DMR with the binding motif for TF_i_, a Gaussian distribution of 21-bp width and 5-bp standard deviation is placed.
3. The TF_i_ binding vector is normalized to sum to 1.
4. Distance between every TF that binds in the DMR is calculated as the absolute value of the difference between every pair of TF binding vectors. This yields a distance matrix of size m_DMR_ × n_DMR_.
5. In order to ensure that results are not biased by TFs that share the same binding motifs or exact same binding locations, TFs are eliminated such that there are no pairs with distance < 0.1. Specifically, in an iterative process, all pairs of TFs with distance < 0.1 are identified. Then one pair is randomly selected, and one of the TFs in the pair is completely eliminated from the distance matrix. This process is repeated until no pairs of TFs have distance < 0.1.
6. Silhouette score (SS) is computed for the distance matrix using TF regulatory cluster memberships.
7. Significance of SS is determined through a permutation test. Specifically, the TF regulatory clustering labels are permuted 10,000 times, each time calculating the corresponding S. *p* value is determined as: [# permutations with SS > actual SS]/10,000.

## Results

### Stimulated gene expression with tetanus toxoid (TT) and German cockroach extract (CR)

**Figure 1:**
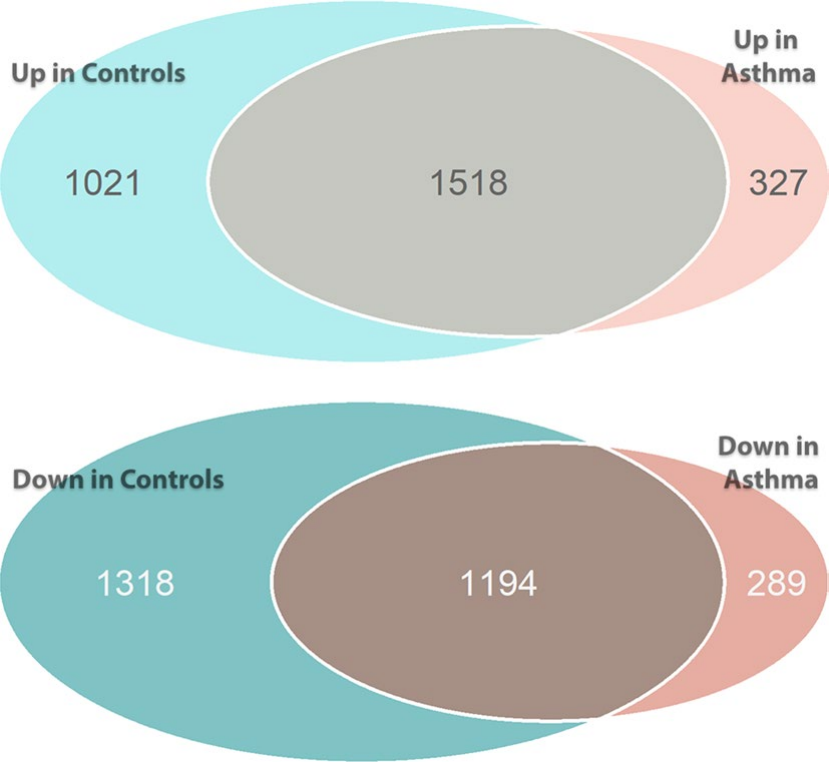
Stimulation of peripheral blood mononuclear cells (PBMCs) with tetanus toxoid (TT) perturbs expression of thousands of genes both in controls and asthma. The number of genes that increase expression (upper Venn diagram) and decrease expression (lower Venn diagram) with TT stimulation are shown for the controls (n = 30) and asthma (n = 19). *DESeq2* was used to perform differential gene expression analysis with FDR-corrected *p* < 0.05.

TT stimulation perturbed expression of thousands of genes, with 5051 genes perturbed in the control group (n = 30) and 3328 genes perturbed in the asthmatic group (n = 19). The discrepancy between number of genes altered in controls vs asthma can be explained by difference in sample size. The genes perturbed in each group were largely overlapping, with 5667 genes total perturbed in at least one group. Specifically, out of 22,426 genes with non-zero read counts for controls, 2539 (11%) were upregulated after TT stimulation and 2512 (11%) were downregulated. In the asthma group (n = 19), out of 22,424 genes with non-zero read counts, 1845 (8.2%) were upregulated after TT stimulation and 1483 (6.6%) were downregulated. These results are summarized in Fig. 1.

Compared to TT stimulation, a much smaller set of genes changed expression with CR stimulation; in the control group (n = 30), 184 (0.8%) were upregulated after CR stimulation and 102 (0.5%) were downregulated. In the asthma group (n = 19), 502 (2.2%) were upregulated after CR stimulation and 304 (1.4%) were downregulated. As previously reported, there were extensive significant interaction effects of group (asthma vs controls) with CR stimulation on gene expression^18^. However, there were no genes with significant interaction effects of group with TT stimulation of PBMCs at age 2.

The genes perturbed by CR stimulation were functionally enriched for biological pathways involved in the allergic response; negative regulation of regulatory T cell differentiation, positive regulation of humoral immune response mediated by circulating immunoglobulin, negative regulation of T-helper Type 1 immune response, T-helper 17 cell lineage commitment. While there was no significant differential gene expression in the children who developed asthma compared to controls in response to TT, this antigen perturbed expression of a much larger set of genes than CR. Our downstream analysis thus aims to characterize immune network changes that may precede the development of asthmatic phenotypes, using TT-elicited gene expression patterns.

### Gene expression modules

WGCNA of the 5667 genes with perturbed expression after TT stimulation in either the control or asthma group yielded 18 gene expression modules. Using Panther gene list analysis, 13 of these modules demonstrate significant pathway enrichment, with a majority of these representing immune pathways. These include an IL1 response pathway, two MHC Class 1 (MHC1) presentation pathways, an immunoglobulin somatic recombination and diversification pathway, a Th2 pathway, two myeloid-mediated immune pathways, and two interferon response pathways. The labels by which we refer to these modules going forward, along with some of their significant GO annotations and the highest centrality genes in each module, are delineated in Table 1. Subsequent analysis was performed only on the 13 modules demonstrating significant enrichments.

**Table 1:** WGCNA modules from tetanus toxoid-stimulated gene expression, with associated gene ontology (GO) annotations and high centrality genes.

Module	GO annotations	Genes with high centrality in STRING PPI network
Mitos-1	Mitotic nuclear division	CDC20, CDK2, BIRC5, PLK1, AURKA
	Sister chromatid segregation	
IL1	Cellular response to IL-1	HSP90AA1, BCL2, CASP3, UBE2N, CHUK
	IL-1-mediated signaling	
	Protein modification by small protein removal	
Mitos-2	DNA replication	CDK1 , PRKCB, PCNA, TOP2A, BRCA1
	Chromosome segregation	
	Cell cycle checkpoint	
Ig_rec	Somatic recombination of immunoglobulin genes involved in immune response	PARP1, MCM5, MCM7, POLA2, H2AFX
	Somatic diversification of immunoglobulins involved in immune response	
	DNA-dependent DNA replication	
MHCI-1	Antigen processing and presentation of exogenous peptide antigen via MHC class I	ACTA2, SF3B3, UBC, TUBG1, BCL3
	Tumor necrosis factor-mediated signaling pathway	
	Regulation of hematopoietic stem cell differentiation	
LPS	Response to LPS	IL6, IL1B, LEP, SOCS3, IL1A
	Response to molecule of bacterial origin	
	Positive regulation of VEGF production	
MHCI-2	Antigen processing and presentation of peptide antigen via MHC class I	STAT3, RUNX3, STAP2, AMER1, PIM2
	Positive regulation of NFkB signaling	
Th2	Humoral immune response mediated by circulating immunoglobulin	PDGFB , ITGB3, MMP1, NBEAL2, CCL13
	B cell mediated immunity	
	Adaptive immune response	
Myel-1	Neutrophil activation involved in immune response	CAT, LPL, TLR4, HSD17B4, ACSL1
	Positive regulation of macrophage derived from foam cell differentiation	
	Positive regulation of monocyte chemotaxis	
	Receptor-mediated endocytosis	
Ifn-1	Response to type 1 interferon	OAS1, ISG15, OASL, MX2, IRF7
	Defense response to virus	
Metab	Lipid catabolic process	INSR, HSPA5, SDC2, PPARG, APOE
Myel-2	Myeloid cell activation involved in immune response	TSPO, RHOA, JUN, RAC1, ICAM1
	Neutrophil degranulation	
	Neutrophil activation	
Ifn-2	Defense response to virus	DDX58, IFIH1, HERC5, STAT1, IFIT1
	Response to interferon-beta	
	Regulation of interferon-alpha production	

**Figure 2:**
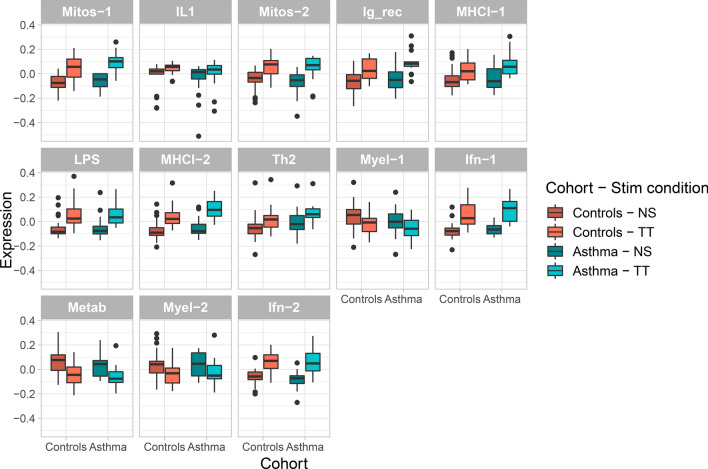
Module eigengenes change significantly with tetanus toxoid (TT) stimulation of peripheral blood mononuclear cells, but demonstrate no group effects. Eigengene expression is displayed for each WGCNA module, separately for controls and asthma, with no stimulation (NS) and TT stimulation of PBMCs. Every module demonstrates a significant effect of stimulation (*p* < 0.01), but none demonstrate a significant group x stimulation effect (*p* < 0.05).

As expected, all 13 modules demonstrated statistically significant effects of stimulation using two-way ANOVA with repeated measures (*p* < 0.01), with all exhibiting increased eigengene expression except for Myel-1, Metab, and Myel-2, which showed decreased expression with TT stimulation (Fig. 2). None of the modules demonstrated significant interaction effects of group (controls vs asthma) with TT stimulation (*p* < 0.05).

### Network connectivity of gene expression modules

**Figure 3:**
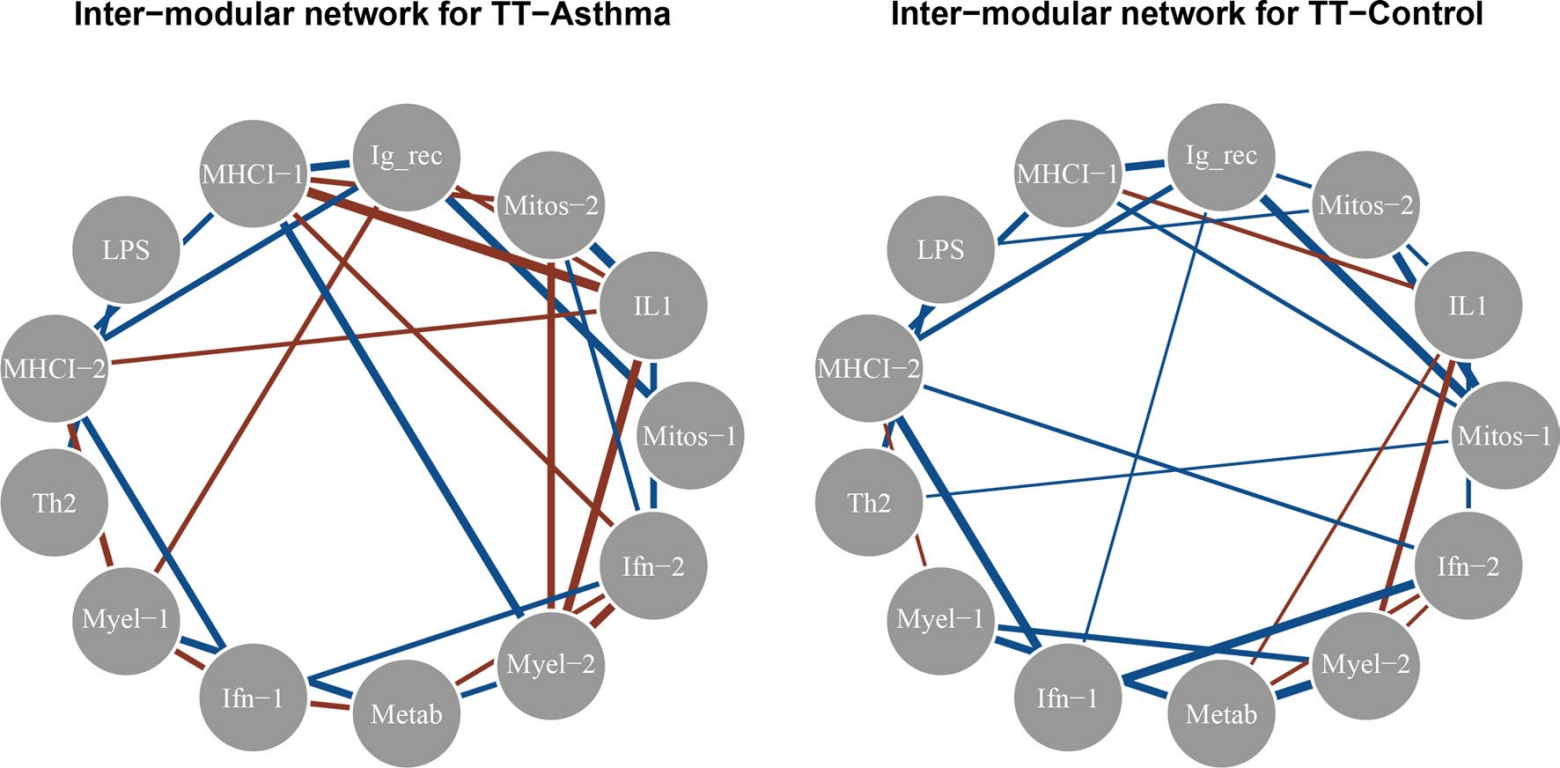
Gene module network differences in asthma are characterized primarily by aberrant negative co-regulation. All significantly positively co-regulated modules (q < 0.05, FDR corrected) are connected with blue edges, while negatively co-regulated modules are connected with red edges. Edge thickness corresponds to strength of correlation.

Both the control and asthma gene expression network demonstrate extensive co-regulation between WGCNA gene expression modules (Fig. 3). The control and asthma networks demonstrate similar overall network structure (the majority of significant edges in each group are shared by both groups). However, there is extensive gain of negative co-regulation in the asthma network compared to the control network, and to a lesser degree, loss of positive co-regulation (Figs. 3; S1).

### Regulatory network alterations

**Figure 4:**
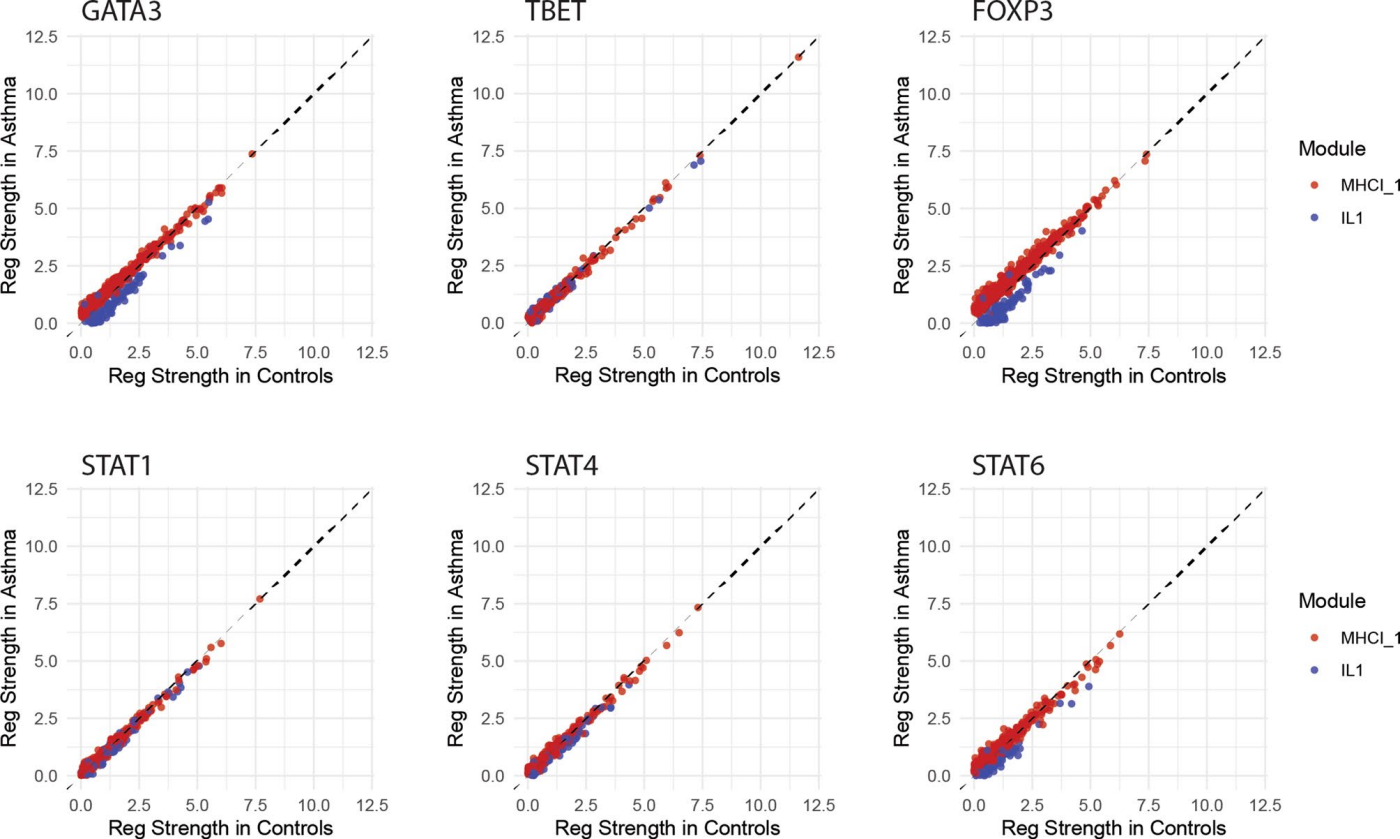
Asthma regulatory networks demonstrate extensive alterations, with both increased and decreased transcription factor (TF) regulation strength. Regulatory strengths outputted from PANDA are displayed for several example TFs in two representative WGCNA modules (MHCI_1 and IL1). The y axis represents regulatory strength in the asthma network while the x axis represents regulatory strength in the control network. Each individual point represents the regulatory strength of the given TF on each of its gene targets in the representative modules. Points above and below the y = x line respectively indicate stronger regulation in asthma relative to controls, and controls relative to asthma.

Extensive regulatory differences were found between the TT-stimulated asthma and control networks using PANDA, with the majority of transcription factors (TFs) demonstrating altered regulation after FDR correction (284 out of 338) (Figure S2). Of the 284 TFs with altered regulation, 135 show weakened regulation, and 149 show strengthened regulation. The magnitude and direction of regulatory shift not only varies by TF, but also varies for a given TF by the WGCNA module membership of the gene targets. Examples of this are shown in Fig. 4 for several representative TFs—GATA3, T-bet, FOXP3, STAT1, STAT4, and STAT6. Two representative WGCNA modules are displayed for demonstrative purposes—MHCI_1, which demonstrated extensive differences of co-regulation in the expression network, and IL1, which demonstrated stronger negative co-regulation with MHCI_1 in the asthma network. GATA3 demonstrates stronger regulation of its targets in the MHCI_1 module in asthma, but weaker regulation of its targets in the IL1 module. FOXP3 and STAT6 demonstrate a similar pattern of altered target regulation, with FOXP3 demonstrating an even more amplified difference between regulation of its targets in these two modules, and STAT6 demonstrating a more subtle difference. In comparison, T-bet, STAT1, and STAT4 regulation of their targets do not appear to significantly differ between the MHCI_1 and IL1 modules (the points lie along the y = x line). This indicates that certain TFs exhibit simultaneous strengthened and weakened regulation of gene targets, and that the direction of regulatory shift depends systematically on target WGCNA module membership.

The two top TFs with the largest median regulatory shift across modules were KDM5B and ARID3A, which have been shown to be important regulators of the epigenome.

### Transcription factor clustering by patterns of regulatory shift

Due to the recognized role of epigenetics in asthma, we hypothesized that broad alterations of the epigenetic landscape might drive the differential transcriptomic and regulatory networks that control the predisposition to disease. TFs bind to regulatory regions of their respective gene targets to activate or repress transcription. Thus, TFs with proximal binding sites on their gene targets will likely be similarly altered by epigenetic alterations of genomic accessibility (i.e. DNA methylation) in these regulatory regions. The presence of broad alterations to the epigenetic landscape could therefore cause shared patterns of “regulatory shift” of TFs that bind at similar locations.

**Figure 5:**
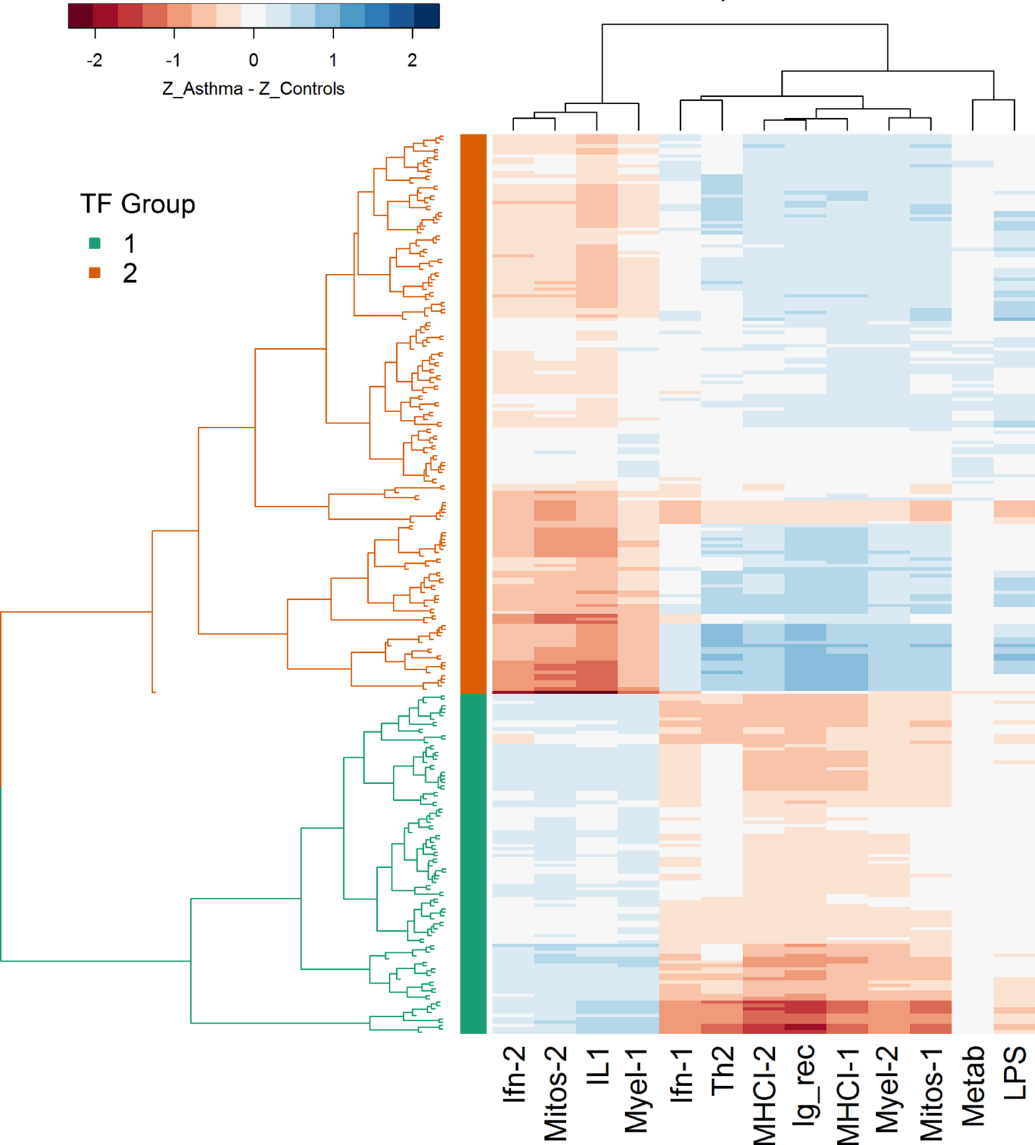
Transcription factors (TFs) cluster into groups based upon the pattern of their regulatory alteration across gene expression modules. A heatmap of the median regulatory shift of targets within each gene expression module (for a given TF: median value of z_Asthma_–z_Control_ across targets within a module). The x axis represents different WGCNA modules, while TFs are represented on the y axis, with branches colored by TF communities from hierarchical clustering. Blue/positive values represent stronger regulatory control in asthma, while red/negative values represent strong regulatory control in controls.

A heatmap of median regulatory shift per TF with each WGCNA module reveals community structure of TFs, where communities of TFs demonstrate shared patterns of regulatory shift across WGCNA modules (Fig. 5). For example, certain TFs show predominantly weaker regulation of targets in Ifn-2, Mitos-2, IL1, and Myel-1 in asthma compared to controls, but stronger regulation of targets in the remaining modules. In contrast, TFs labeled by the green bar on the y axis show an inverse pattern of dysregulation; these TFs show predominantly stronger regulation of targets in Ifn-2, Mitos-2, IL1, and Myel-1 in asthma compared to controls, but weaker regulation of targets in the remaining modules. Hierarchical clustering of TFs based on these median shifts per module yields an optimal cluster number of two TF groups based on the maximum silhouette score.

### Binding locations of transcription factor groups

**Figure 6:**
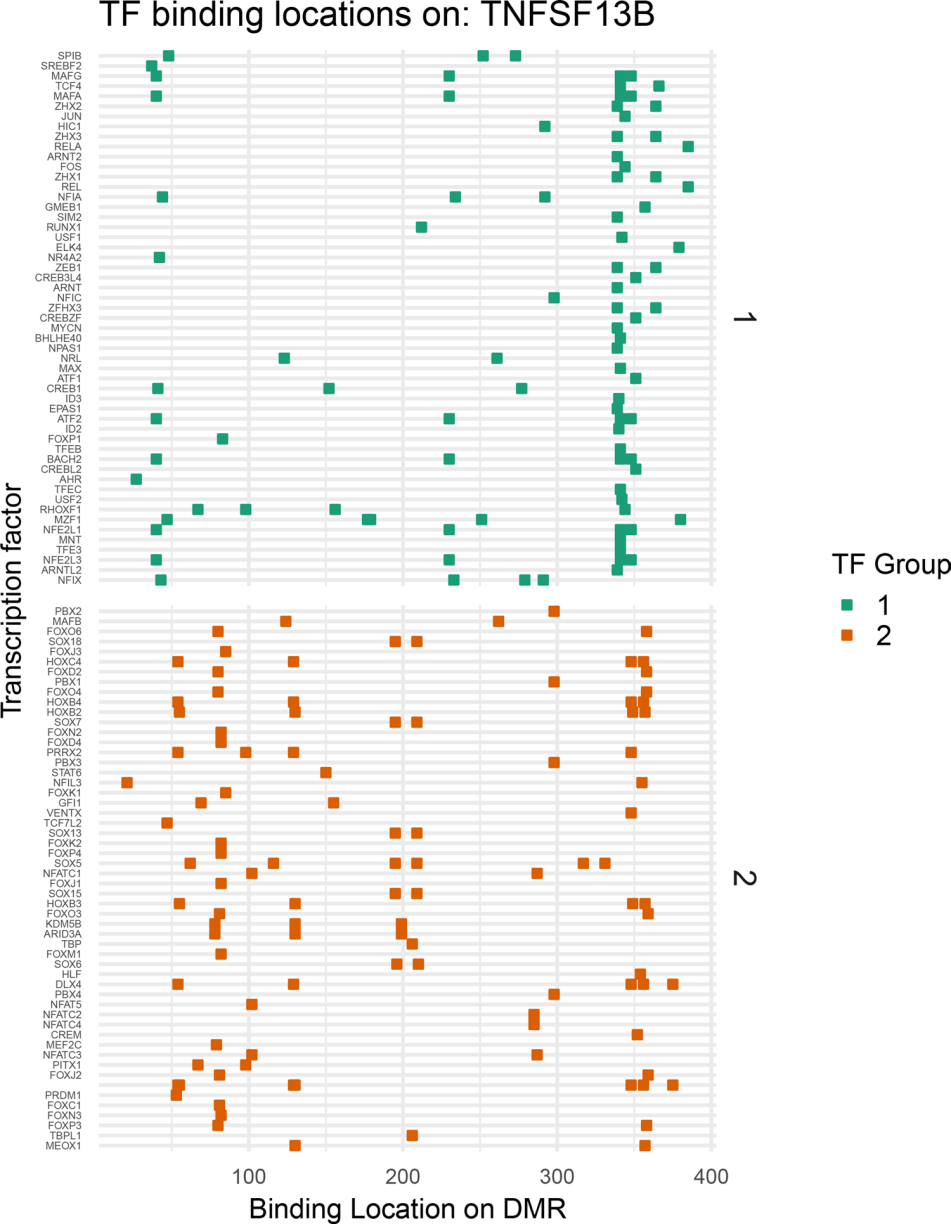
Transcription factors (TFs) within the same regulatory communities bind in similar locations on differentially methylated regions (DMRs). Binding locations of TFs with binding motifs (based on 80% of maximum confidence) in a DMR in TNFSF13B. The TFs are separated based upon their regulatory cluster memberships as defined in Fig. 4.

**Figure 7:**
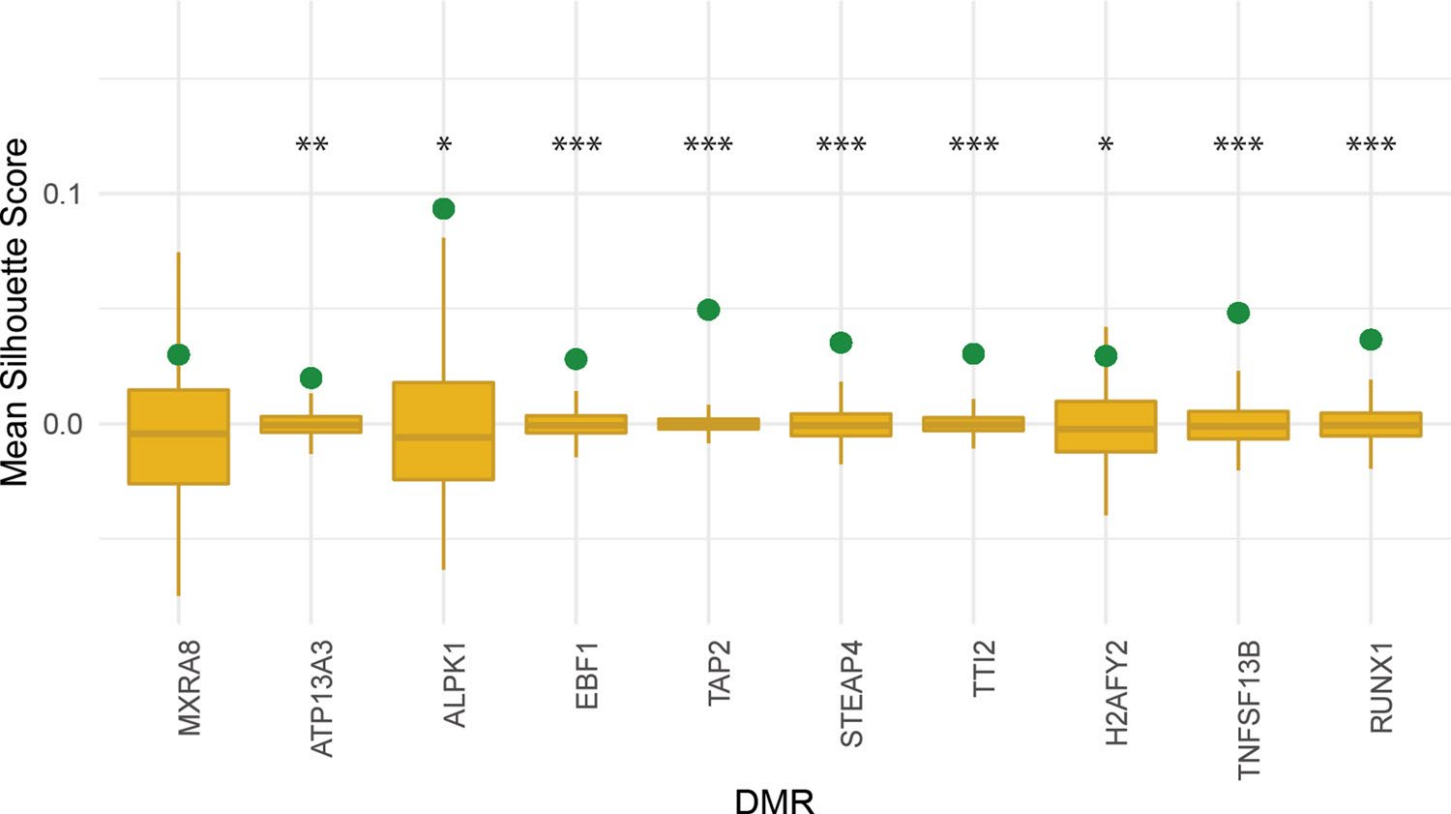
Regulatory communities of transcription factors (TFs) exhibit significant clustering based on binding distances within differentially methylated regions (DMRs). The genes on the X axis represent all DMRs from the Reese et al. meta-analysis of CBMCs, which demonstrated perturbation of expression from tetanus toxoid (TT) stimulation. The green dot represents the mean silhouette score (SS) calculated from the binding location distance matrix with TF regulatory clusters. The gold points and density plots represent the null distribution of mean SS from each of 10,000 permutations of the regulatory cluster labels. Asterisks represent statistical significance *p < 0.05; **p < 0.01; ***p < 0.001.

TFs that cluster into the same communities based upon regulatory shift tend to have proximal binding locations to one another in DMRs (Figs. 6, 7). Binding locations of TFs with motifs in a differentially methylated region of TNFSF13B (encodes B-cell activating factor (Baff)) are shown to illustrate the proximal binding of TFs within the same communities (Fig. 6). The TFs are separated based upon their regulatory community. TFs within the same community bind more closely to one another than TFs between regulatory communities. The similarity of binding locations within communities suggests that shared patterns of dysregulation may be driven by permissive or repressive epigenetic changes at the level of the target genes.

The mean SS from the binding location distance matrix using TF regulatory cluster memberships is displayed for all 10 DMRs (Fig. 7). For the majority of DMRs, the mean SS is significantly higher (*p* < 0.001) than the null distribution of silhouette scores (SS) generated from permutation of TF regulatory cluster labels. Thus, regulatory communities of TFs cluster based upon their binding distances within DMRs.

## Discussion

We characterize asthmatic poise by probing gene–gene interactions and inferring epigenetic alterations. Specifically, we identify significantly altered interactions in our gene expression networks. We also demonstrate that these altered expression networks can be explained by regulatory differences, and provide evidence that broad epigenetic alterations cause the downstream disruption of these networks. In summary, gene network investigation powered by associations of stimulated gene expression across subjects allows us to uncover immune imbalances that precede clinical diagnosis of asthma.

The differential gene networks elucidated with tetanus toxoid (TT) stimulation suggest that broad immune imbalances prime allergic sensitization. Prior transcriptomic studies of childhood asthma have investigated subjects who already have a disease diagnosis, and have primarily investigated differential gene expression as opposed to differential gene interactions. Significant differential gene expression in response to German cockroach extract (CR), but not TT, was previously reported in this cohort^18^. However, this was only found in children with both CR sensitization and asthma by the age of 7, and not in children without CR sensitization. We posit that the differential response to CR, which perturbs a much more limited set of genes (~ 800 for asthma and ~ 300 for controls, versus ~ 5000 for TT stimulation in both groups), represents an allergic response to CR. In contrast, TT elicits more widespread non-allergen-specific stimulation of immune responses in both groups, allowing us to uncover network states poised for allergic sensitization. Since allergic asthma can be triggered by diverse allergens depending on individual exposure, it is valuable to identify non-exposure-dependent differential immune responses (i.e. to TT) that characterize allergic predisposition.

While we expected to find regulatory network alterations, the unexpected findings here are the uncovered patterns of dysregulation. It is particularly interesting that a given TF can exhibit strengthened regulation of some targets in the asthma group compared to controls, but weakened regulation of other targets. Even more striking, the direction and magnitude of change varies systematically depending upon the WGCNA module membership of the target gene. This finding, combined with the demonstrated shared patterns of dysregulation amongst communities of TFs (Fig. 5) and proximal binding locations of TFs with the same community, support the idea that broad epigenetic alterations perturb regulatory and expression networks. The absence of statistically detectable differential gene expression, concurrent with regulatory network alterations, is consistent with previously indicated epigenetic mechanisms of asthmatic poise. A prior study in a murine model of asthma found minimal gene expression differences in dendritic cells from asthma-at-risk neonates compared to control mice, despite the presence of extensive genome-wide methylation differences^28^. Substantial differential gene expression became evident only upon allergen sensitization, primarily among transcripts that showed epigenetic alterations at birth. Taken together, these suggest a paradigm in which subtle but widespread changes to the epigenetic landscape poise the immune system for allergic sensitization. Subsequently, allergic sensitization leads to enhanced epigenetic modifications, differential gene expression and cytokine elaboration, and phenotypic disease manifestation.

This paradigm is further supported by the TFs that we found to demonstrate the most altered regulation across modules; ARID3A and KDM5B have both been implicated as important players in epigenetic control. ARID3A is required for hematopoietic stem cell differentiation and B cell development, and has been shown to suppress somatic cell reprogramming^29^. ARID3A also activates transcription of the immunoglobulin heavy chain (IgH) by altering chromatin accessibility to the IgH enhancer^30^. KDM5B is a histone demethylase, a critical regulator of genome stability required for efficient DNA double-strand break repair, and has been shown to be enriched at DNA-damage sites after ionizing radiation and endonuclease treatment^31^. It represses expression of genes involved in immune cell proliferation and migration, and may cooperate with histone deacetylase in repression of gene expression^32,33^.

There is evidence of epigenetic differences in asthmatics, with genome-wide methylation studies demonstrating predominantly permissive methylation differences as early as birth^16^. However, results of epigenetic studies have not established robust associations with downstream gene expression. These prior studies have focused on DNA methylation, and thus do not capture the full landscape of epigenetic alterations^34^. Studies investigating histone modifications have provided more mechanistic insight into epigenetic changes in asthma. The ratio of histone deacetylase to histone acetylase is lower in lung samples of asthmatics, correlates with disease severity, and corrects with treatment^10–13^. Further, HDAC inhibition in ex vivo memory T cells results in strongly elevated Th2 cytokine production and reduced Th1 cytokine production during immune recall response^14^. Interestingly, this shift in Th2/Th1 cytokine responses is driven by elevation of the master Th2 regulator GATA3, without change in the expression level of the corresponding Th1 regulator T-bet. These findings are consistent with our finding of strong regulatory differences in GATA3 but not T-bet as a function of gene module membership (Fig. 4). GATA3 interacts with HDACs and methyltransferases to produce suppressive changes at Th1 loci, and with HAT to create permissive changes at Th2 loci^35–37^. It binds to its own regulatory elements, positively regulating its own expression^38^. These findings further expand upon epigenetic theories of early atopic predisposition, in which positive feedback mechanisms progressively destabilize immune balance, ultimately producing measurable differential gene expression and asthmatic phenotypes.

This study is limited by lack of epigenetic data to validate the inferred alterations, as well as by the pooled cell populations, which make it challenging to identify key cellular players in the altered gene interactions. Future investigation will benefit from simultaneous collection of transcriptomic and epigenomic data from separated cell populations or single cell analyses. Collection of epigenomic data using a method such as Assay for Transposase-Accessible Chromatin using sequencing (ATAC-Seq) will facilitate validation and complementary characterization of regulatory relationships. Additionally, it may allow for diagnosis or prognosis of individual subjects using epigenomic fingerprints of altered accessibility at regulatory regions of DNA. Our present transcriptomic network analyses allow us to identify group-level network differences. However, it would be challenging to perform individual diagnosis based upon this framework, since construction of subject-level networks would require several datasets per subject, or would rely on unstable statistical inference methods. It will also be of interest in the future to determine whether altered expression and regulatory networks can be discerned even earlier in life (e.g. by studying cord blood mononuclear cells (CBMCs)), as epigenetic influences begin in utero.

In conclusion, we have described a novel framework to characterize transcriptomic network alterations, shown that gene network dysregulation can be detected in atopically predisposed individuals long before clinical asthma diagnosis, and provided evidence that these atopically primed networks are a result of widespread alterations of the epigenetic landscape. Our approach indicates the potential to identify development of allergic disease including asthma prior to clinical diagnosis.

## Supplementary information

Supplementary Legends.

Supplementary Figure 1.

Supplementary Figure 2.

Supplementary Table 1.
