# Supplementary material for: Immune network dysregulation precedes clinical diagnosis of asthma
Source: Sci Rep. 2020 Jul 30;10:12784. doi: 10.1038/s41598-020-69494-x (PMC7393349; doi:10.1038/s41598-020-69494-x)
Supplement: Supplementary file 1 — Supplementary Legends. [file 41598_2020_69494_MOESM1_ESM.docx]

**IMMUNE NETWORK DYSREGULATION PRECEDES CLINICAL DIAGNOSIS OF ASTHMA**

Yi-Shin Chang^1,2^, Benjamin Turturice^1,3^, Cody Schott^1,3^, Patricia Finn^1,2,3^*, David Perkins^2,4,5^*

**Affiliations:**

1. Department of Medicine, Division of Pulmonary, Critical Care, Sleep, and Allergy, University of Illinois at Chicago College of Medicine, Chicago, IL

2. Department of Bioengineering, University of Illinois at Chicago College of Medicine, Chicago, IL

3. Department of Microbiology and Immunology, University of Illinois at Chicago College of Medicine, Chicago, IL

4. Department of Medicine, Division of Nephrology, University of Illinois at Chicago College of Medicine, Chicago, IL

5. Department of Surgery, University of Illinois at Chicago College of Medicine, Chicago, IL

*These authors contributed equally.

**Figure S1: The asthma gene module network demonstrates several aberrant negatively co-regulated modules**. R values from Pearson correlations between the eigengenes of every pair of modules are displayed for the asthma network on the y axis, and control network on the x axis. Points are colored by statistical significance of the correlations, with FDR correction across all pairs of tested modules. The solid line is y=x, representing the line of equal edge strengths between controls and asthma, and the dotted line is a linear regression with 95% confidence interval shading.

**Figure S2: Transcription factors (TF) exhibit both significantly strengthened and significantly weakened regulation in the asthma regulatory network**. A histogram of t-statistics for each TF in the regulatory network is shown. The t-statistic for a given TF was calculated by computing the pairwise student T-test of the regulatory strength across all gene targets in the asthma regulatory network (generated by PANDA) compared to the regulatory strength across all gene targets in the control regulatory network.

**Table S1: Transcription factors (TF) used in regulatory network construction with PANDA (Passing Attributes between Networks for Data Assimilation).** The listed transcription factors had DNA binding profiles listed in JASPAR, a transcription factor database. Their position frequency matrices (PFMs) were thus mapped to the promoter regions of each target gene to obtain an initial regulatory network inputted to PANDA.
