# Supplementary figures and images for: Immune network dysregulation precedes clinical diagnosis of asthma
Source: Sci Rep. 2020 Jul 30;10:12784. doi: 10.1038/s41598-020-69494-x (PMC7393349; doi:10.1038/s41598-020-69494-x)

Rval - Asthma Network

Rval - Ctrl Network

Edge significance

- Both
- Ctrl only
- Asthma only
- Neither

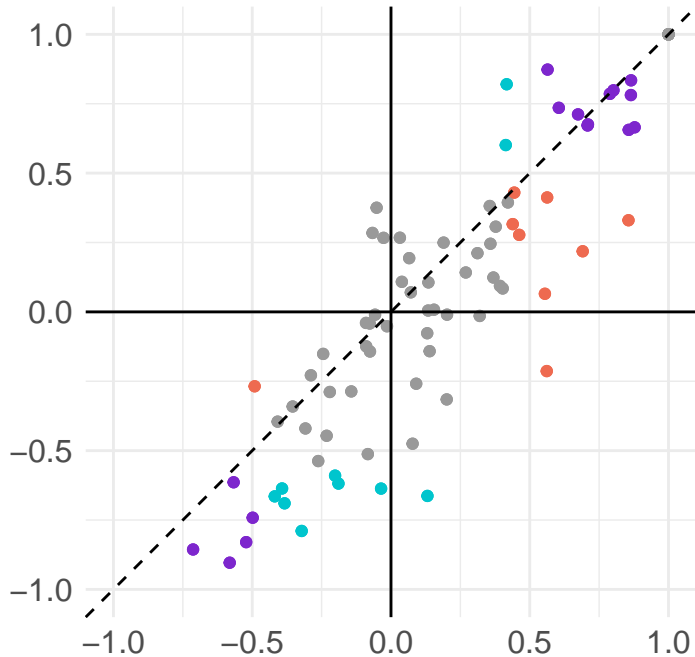

Supplement: Supplementary file 2 — Supplementary Figure 1. [file 41598_2020_69494_MOESM2_ESM.pdf]

# Regulatory differences of Controls vs Asthma per TF

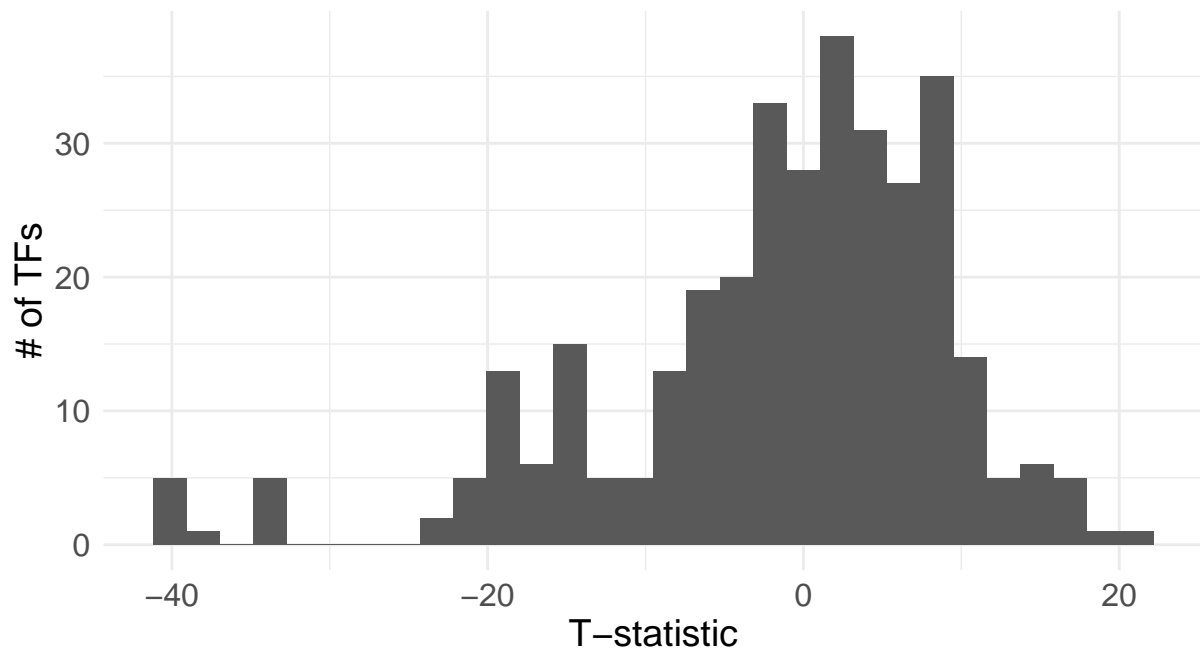

Supplement: Supplementary file 3 — Supplementary Figure 2. [file 41598_2020_69494_MOESM3_ESM.pdf]
